# Comparative analysis of the ultrastructure and adhesive secretion pathways of different smooth attachment pads of the stick insect *Medauroidea extradentata* (Phasmatodea)

**DOI:** 10.3762/bjnano.15.52

**Published:** 2024-05-29

**Authors:** Julian Thomas, Stanislav N Gorb, Thies H Büscher

**Affiliations:** 1 Functional Morphology and Biomechanics, Institute of Zoology, Kiel University, Am Botanischen Garten 9, 24118 Kiel, Germany

**Keywords:** adhesion, arolium, biomechanics, euplantulae, friction, functional morphology, tarsal secretion

## Abstract

The mechanism by which insects achieve attachment and locomotion across diverse substrates has long intrigued scientists, prompting extensive research on the functional morphology of attachment pads. In stick insects, attachment and locomotion are facilitated by two distinct types of smooth cuticular attachment pads: the primary adhesion force-generating arolium and the friction force-generating euplantulae. They are both supported by an adhesive secretion delivered into the interspace between the attachment pads and the substrate. In this study, we analysed and compared internal morphology, material composition and ultrastructure, as well as the transportation pathways in both adhesive organs in the stick insect *Medauroidea extradentata* using scanning electron microscopy, micro-computed tomography, light microscopy, and confocal laser scanning microscopy. Our observations revealed structural differences between both attachment pads, reflecting their distinct functionality. Furthermore, our results delineate a potential pathway for adhesive secretions, originating from exocrine epidermal cells and traversing various layers before reaching the surface. Within the attachment pad, the fluid may influence the viscoelastic properties of the pad and control the attachment/detachment process. Understanding the material composition of attachment pads and the distribution process of the adhesive secretion can potentially aid in the development of more effective artificial attachment systems.

## Introduction

Throughout their evolutionary timeline, insects evolved various surfaces interacting with the environment. These include friction-based adhesive organs, which are essential for locomotion by generating frictional and adhesive forces [1–4]. Two morphologically different friction-based adhesive principles convergently emerged in insects multiple times: hairy and smooth adhesive organs [5–7]. Both principles are used for multiple functions from locomotion [8–9] to attachment during copulation [10] and predator resistance [11].

To fulfil their functions, smooth attachment pads need to enhance the actual contact area between the pad and the substrate for the realisation of efficient attachment due to adhesion and friction forces [3,9,12–14]. Smooth attachment pads have independently evolved in most large insect groups, possessing multiple specialized types of pads on the same leg that are adapted to attachment through the division of labour by preferably generating more adhesion or friction [5]. Adhesive secretion in the contact zone between the attachment pad and substrate supports the functionality of the pads [15].

The adhesive secretion can fill the gaps in the substrate roughness and thereby increase the contact area [14,16–19]. It can aid in the enhancement of viscous and capillary forces further increasing the attachment strength [9,14,20–24]. The adhesive secretion can be essential for the self-cleaning mechanism by binding smaller contamination particles together into larger complexes for easier removal [25–26]. It can also improve attachment to surfaces with different surface chemistry by mediating between the two surfaces in contact [27–28]. The lipid-containing pad secretion protects the insect from additional water loss through the thin-walled attachment pads [29] and assists in chemical communication [30].

The tarsal secretion can facilitate these functions due to its chemical composition and the resulting physical properties. Chemical analyses of the tarsal fluid revealed that its composition differs between different insect groups but mostly contains water-soluble and lipid-soluble substances [31–35] creating lipid droplets in an aqueous fluid [27,36] or hydrophilic nanodroplets embedded in an oily continuous phase [23,37]. Additionally, the tarsal secretion could be a mixture of multiple substances that are present in varying mixture ratios, which would also influence its properties and thus its functions [38]. Secretion with more long-chain carbons and higher branching bonds is more viscous and would potentially exert stronger viscous forces [39–40].

The functional differentiation of the smooth attachment pads likely arises from differences in the ultrastructure and material composition of the pad types and is potentially supported by possible differences in the produced tarsal secretion. Despite extensive research on the attachment capabilities and the ultrastructure of the different attachment pads in various insect groups (for example, Coleoptera [5], Hemiptera [41], Diptera [42–43], Orthoptera [5,20,44], and Blattodea [45]), knowledge on the differences in the internal ultrastructure and fluid transportation between different types of smooth attachment pads located on the same tarsus is scarce, especially in Phasmatodea. Recent investigations of the ultrastructure and material properties of the smooth tarsal attachment pads of phasmids complement our information on the morphology of the droplets [38], biomechanics of their attachment performance [28,46–51], and the complementarity of the two pad types [47,52–53].

In this study, we compare the ultrastructure and material composition of the two smooth tarsal (euplantulae) and pretarsal (arolium) attachment pads of the stick insect *Medauroidea extradentata* (Brunner von Wattenwyl, 1907), focusing on their functional differences as well as on the tarsal secretion production pathways. It was previously shown that the euplantulae are used to generate stationary attachment forces and propulsion (frictional pad) and the arolium to generate adhesion forces (adhesion pad) [52,54]. *M. extradentata* was selected here due to its relatively large adhesive organs that bear no further surface microstructures [47,55–56] and because the droplet morphology of its tarsal secretion has been recently analysed [28,38,47,55–56].

Combining different imaging techniques, including scanning electron microscopy (SEM), confocal laser scanning microscopy (CLSM), histological staining of longitudinal and cross sections (toluidine blue and Cason), and micro-computed tomography (µCT), our investigation of the arolium and euplantulae of the stick insect *M. extradentata* addresses the following questions: (1) Are there structural and material differences between the tarsal frictional pads (euplantulae) and the pretarsal adhesion pads (arolia)? (2) Where is the adhesive secretion produced and stored? (3) How many different types of exocrine cells producing pad secretions do exist? (4) How is the adhesive secretion transported from the production site to the pad surface? The results could enhance our overall comprehension of the functionality of the two smooth attachment organs, euplantulae and arolium, also shedding light on the fluid production and transportation processes in different smooth pads of Phasmida.

## Materials and Methods

### Animal

**Figure 1:**
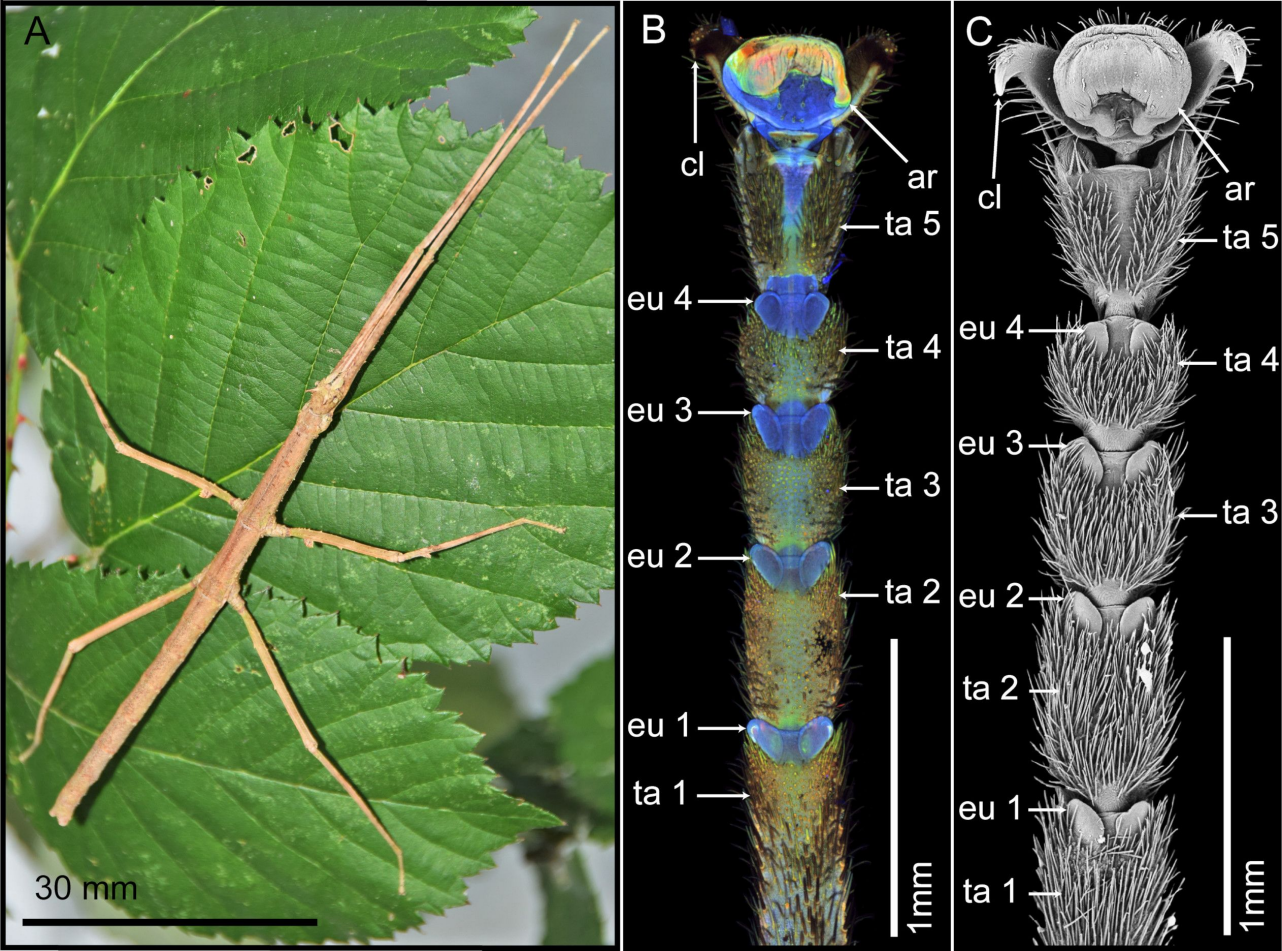
*Medauroidea extradentata* and its tarsal structures. Example images of the animals used in the experiments and their tarsi. (A) Female. (B) CLSM maximum intensity projection of the tarsus. (C) SEM image of the tarsus. ar = arolium; cl = claw; eu 1–5 = euplantulae 1–5; ta 1–5 = tarsomeres 1–5. Figure 1C was adapted with permission of The Company of Biologists Ltd., from [28] (“Influence of surface free energy of the substrate and flooded water on the attachment performance of stick insects (Phasmatodea) with different adhesive surface microstructures” by J. Thomas et al., *J. Exp. Biol.*, vol. 226, issue 2, jeb244295, © 2023); permission conveyed through Copyright Clearance Center, Inc. This content is not subject to CC BY 4.0.

We used the phasmid species *Medauroidea extradentata* (Brunner von Wattenwyl, 1907) (Figure 1A), because of the availability of livestock and the presence of the functional morphology data on its tarsal attachment system [28,46–49].

Individuals were obtained from the laboratory cultures of the Department of Functional Morphology and Biomechanics (Kiel University, Germany). The insects were fed with blackberry leaves *ad libitium* and kept in a regular day and night cycle. Only adult female individuals were selected. The animals were kept with blackberry leaves in clean hard plastic boxes to reduce contamination of the attachment pads.

### Light microscopy

Two tarsi of adult female *M. extradentata* were dissected into five tarsomeres. The proximal four tarsomeres bear one euplantula each, whereas the fifth tarsomere additionally carries the pretarsus including the arolium (see Figure 1B, Figure 1C). The five tarsomeres were fixed in 2.5% glutaraldehyde in (pH 7.4) phosphate-buffered saline (PBS) for 24 h, washed two times in PBS for 30 min each, fixed in 1% aqueous OsO_4_ for 1 h, and washed two times in double-distilled water, for 30 min each. After fixation, the samples were dehydrated using an ascending ethanol series from 30% to 100% (each step for 20 min). All steps were performed on a shaker and at 4 °C. For the last step, the samples were embedded in Epon 812 (Glycidether 100; Carl Roth GmbH, Karlsruhe, Germany) and polymerized at 60 °C for 48 h.

The embedded samples were cut into semi-thin sections of 0.2–1.0 µm using a Leica EM UC7 ultramicrotome (Leica Microsystems GmbH, Wetzlar, Germany) (at 21.5 °C room temperature), mounted on polylysine-covered glass slides (Gerhard Menzel GmbH, Braunschweig, Germany) and stained with toluidine blue or Cason’s triple stain (Romeis 2010). Toluidine blue is a basic metachromatic dye, which selectively stains basophilic tissue components and has a high affinity to acidic tissue (nucleic acids are stained blue and polysaccharides purple). Previous experiments have also shown that the dye stains soft parts of the cuticle dark blue, and sclerotized parts of the cuticle light blue. In addition, the blue colour intensity corresponds to the relative electron density of the tissue in TEM [57–59].

Cason’s triple stain allows for the differentiation of differently sclerotized regions from brown over orange to yellow (with a decreasing degree of sclerotization) to resilin-bearing regions stained from violet to pink [60–61].

For staining with toluidine blue, the glass slides were incubated with 0.1% toluidine blue solution for 2 min and rinsed using a stream of distilled water. Cason’s triple stain (consisted of 1 g of phosphotungstic acid, 2 g of orange G, 1 g of aniline blue, and 3 g of acid fuchsine, dissolved in 200 mL of distilled water [60–61]. Cason stain was applied onto the glass slides for 5 min at 60 °C and rinsed with 70%–100% EtOH and tap water.

The stained samples were observed using a light microscope (Zeiss Axioplan, Carl Zeiss Microscopy GmbH, Jena, Germany) with 40× and 100× lenses. The images were processed using Adobe Photoshop (version CS6; Adobe Systems Inc., San Jose, CA, USA).

### Scanning electron microscopy

Tarsi of *M. extradentata* were cut from adult females and fixed in 2.5% glutaraldehyde in PBS for 24 h. Then, they were washed two times with PBS for 30 min and two times with double-distilled water for 30 min each. Afterwards, the samples were dehydrated in an ascending ethanol series. Each step was performed on ice (4 °C) and on a shaker. Afterwards, the samples were critical point dried (Leica EM CPD300, Leica, Wetzlar, Germany). Then, the dry pretarsal arolium and tarsal euplantulae were dissected at the centre using two fine tweezers to achieve a clean breaking edge. The samples were mounted on aluminium stubs and sputter-coated with a 10 nm layer of gold–palladium (Leica BalTec SCD 500, Leica, Wetzlar, Germany). The images were obtained using a scanning electron microscope (TM 3000, Hitachi High-Technologies Corp, Tokyo, Japan) at 3 kV acceleration voltage. The recorded images were stitched, merged, and processed using the software Photoshop CS6 (Adobe Systems Inc., San Jose, CA, USA).

### Confocal laser scanning microscopy

Whole tarsi and cross sections of the pretarsal (arolium) and tarsal (euplantulae) attachment pads of adult female stick insects *M. extradentata* were analysed using CLSM. Fresh tarsi of *M. extradentata* were cut off, directly placed in 100% Triton X-100 (Sigma-Aldrich Chemie GmbH, Steinheim, Germany) for 30 min, and then transferred to glycerine. To analyse the entire tarsus, it was directly transferred onto a glass slide and mounted with a coverslip (thickness = 0.170 ± 0.005 mm, refractive index = 1.52550 ± 0.00015, Carl Zeiss Microscopy GmbH, Jena, Germany). For the cross sections of arolium and euplantulae, the attachment pads were cut with a carbon blade and individually transferred onto a glass slide and mounted with a coverslip (specifications as above).

For analysis, a confocal laser scanning microscope (Zeiss LSM 700, Carl Zeiss Microscopy GmbH, Jena, Germany) and four stable solid-state lasers (wavelengths 405, 488, 555, and 639 nm in combination with the respective bandpass and longpass emission filters BP420–480, LP490, LP560, LP640 nm) were used.

The whole tarsi were visualised with a 5× lens (Zeiss Plan-Apochromat, air immersion, numerical aperture = 0.16, Carl Zeiss Microscopy GmbH, Jena, Germany) and the cross sections of the attachment pads with a 20× lens (Zeiss Plan-Apochromat, air immersion, numerical aperture = 0.17, Carl Zeiss Microscopy GmbH, Jena, Germany). Maximum intensity projections were created using the ZEN 2008 software (https://www.zeiss.de/mikroskopie) and subsequently, the contrast and brightness were adjusted in Adobe Photoshop (version CS6; Adobe Systems Inc., San Jose, CA, USA). Three colours: red, green, and blue were assigned according to the emitted autofluorescence wavelength representing to some extent the degree of sclerotization. Red represents the highest sclerotization degree, green – the medium one, and blue – the lowest one (see Figure 1B).

### Micro-computed tomography

A whole tarsus of an adult female *M. extradentata* was cut off at the base of the tibia, directly fixed in 2.5% glutaraldehyde in PBS, and washed in PBS. For the preparation of the µCT scan, the tarsus was dehydrated with an ascending EtOH sequence at 4 °C on a shaker, and subsequently critical point dried using Leica EM CPD300 (Leica, Wetzlar, Germany). The tarsus was scanned using a Skyscan^®^1172 µCT (Bruker micro‐CT; CT‐scanner settings: X‐ray source: 40 kV, 250 μA, 360 rotation, 0.2 rotation step, 10 frames averaging, and 10 random movements), reconstructed in Nrecon^®^1.0.7.4 (Bruker micro‐CT, Billerica, MA, USA), segmented with Amira^®^6.2 (Thermo Fisher Scientific, Waltham, MA, USA), and visualized with the open-source 3D creation software Blender 2.82a (Blender Foundation, Amsterdam, Netherlands) and Affinity Designer (Serif, Nottingham, UK).

## Results

### Tarsal structure

The structure of the tarsus of *M. extradentata* was observed using CLSM and SEM (Figure 1B,C). It comprises five tarsomeres (ta 1–5) and the pretarsus. Tarsomeres one to four (ta 1–4) each bear a pair of euplantulae (eu 1–4) at their distal ends. The pretarsus features the arolium (ar) situated between a pair of claws (cl). The euplantulae, the cuticle between them, and the arolium bear a rather smooth surface structure. The remaining surface of the tarsomeres, where no attachment pads are situated, is covered with setae (Figure 1C). The CLSM images revealed that both types of attachment pads and the cuticle between the euplantulae and between the tarsomeres show a low degree of sclerotization (blue coloration). In contrast, the cuticle of the remaining tarsomeres has a higher degree of sclerotization (green/yellow coloration). Notably, the distal ventral region of the arolium displays a relatively higher degree of sclerotization (green/yellow coloration). Additionally, red coloration is visible inside the arolium; however, this does not correspond to the cuticle, but presumably to the glandular tissue of the arolium (Figure 1B).

### Arolium structure

**Figure 2:**
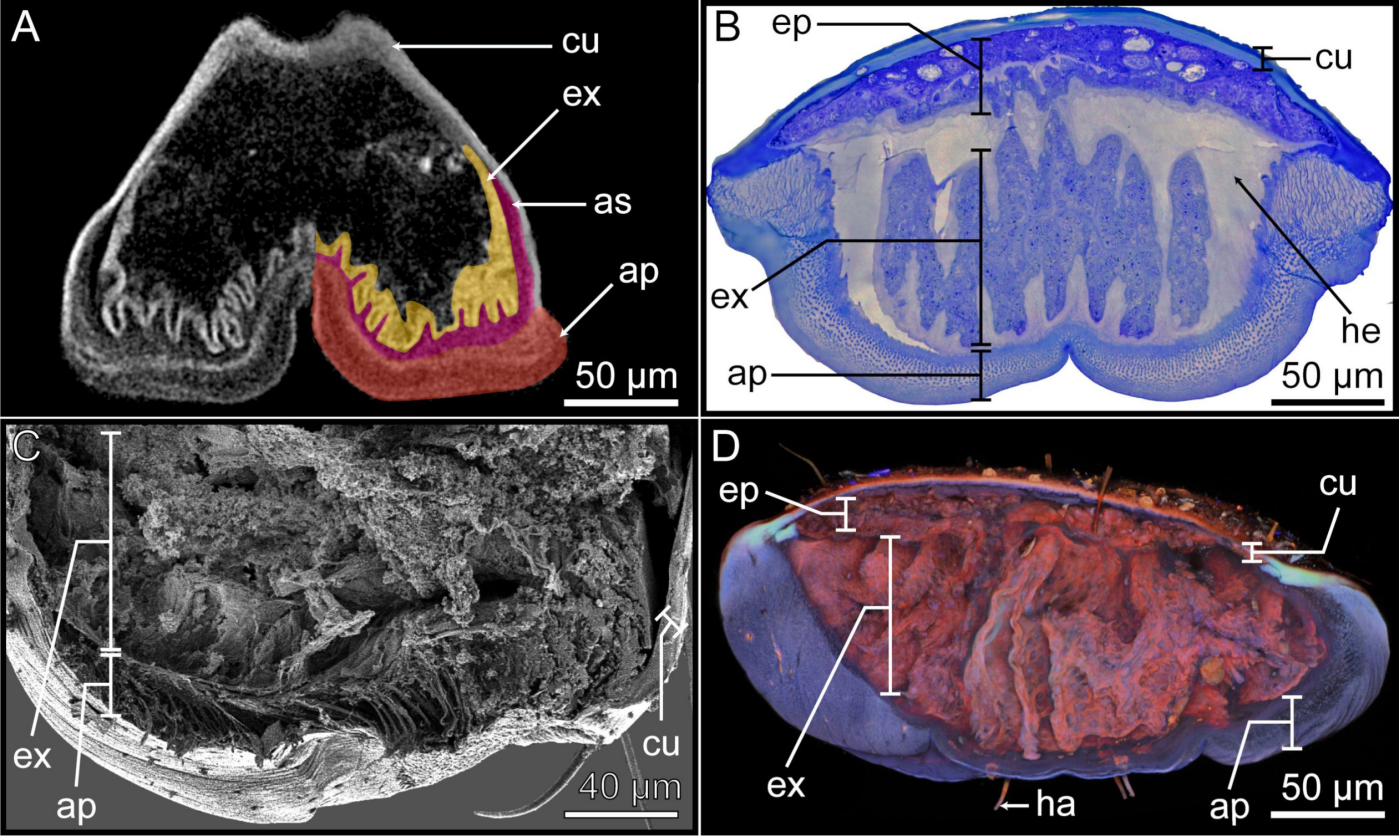
Sections of the arolium visualized with different imaging techniques. The internal ultrastructure of the arolium was visualized using four different methods, which show the different layers and highlight their morphological and structural characteristics. The following methods were used: (A) µCT. (B) Cross section stained with toluidine blue, light microscopy. (C) SEM. (D) CLSM. For images (C) and (D) the arolium had to be dissected. All images are similarly positioned: the ventral side of the arolium is located at the bottom of the picture. Ap = attachment pad; as = adhesive secretion reservoir; cu = cuticle; ep = epidermal cells; ex = exocrine cells; ha = hair/seta; he = hemolymph.

The pretarsus of *M. extradentata* is 500 µm wide and 400 µm long. The ventral face of the arolium consists of a thickened layer of fibrous cuticle composing the actual smooth attachment pad (ap) [1]. Toluidine blue staining resulted in a blue hue of the attachment pad, indicating the presence of a meshed network of flexible cuticle fibres within the attachment pad (Figure 2B). This coarse meshed-fibre structure was also observed in SEM (Figure 2C). In addition, using CLSM, the attachment pad structure exhibited a low degree of sclerotization indicating a presumably soft cuticle (Figure 2D). Internally, the main part of the arolium consists of a large epithelium, recognizable by the light hue of the toluidine blue staining. The epithelium mainly consists of exocrine cells (ex) which display a large surface area towards the hemolymph due to irregular protrusions (Figure 2B). These evaginations are also visible in the µCT cross sections as radio-dense layers (Figure 2A). The exocrine cells exhibited a mixed red/blue signal in CLSM (Figure 2D) and appeared densely packed in the SEM sections (Figure 2C). The exocrine cells are likely surrounded by the hemolymph (he), which appeared yellowish when stained with toluidine blue (Figure 2A).

On the back of the arolium, epidermal cells (ep) are present, separated from the exocrine cells by the hemolymph (Figure 2B). These epidermal cells were stained in a relatively darker hue by toluidine blue (Figure 2B) and displayed a reddish fluorescence signal in CLSM (Figure 2D). However, they were not visible in the µCT cross sections (Figure 2A).

The arolium exhibits a sclerotized cuticle (cu) on its dorsal side. The sclerotized cuticle is composed of two layers, the inner layer showing light blue staining by toluidine blue (Figure 2B) and a light red fluorescence signal in CLSM (Figure 2D), while the outer layer is stained dark blue by toluidine blue (Figure 2B) and shows a dark red fluorescence signal in CLSM (Figure 2D). Both layers show radiodensity in µCT (Figure 2A).

### Arolium ultrastructure

**Figure 3:**
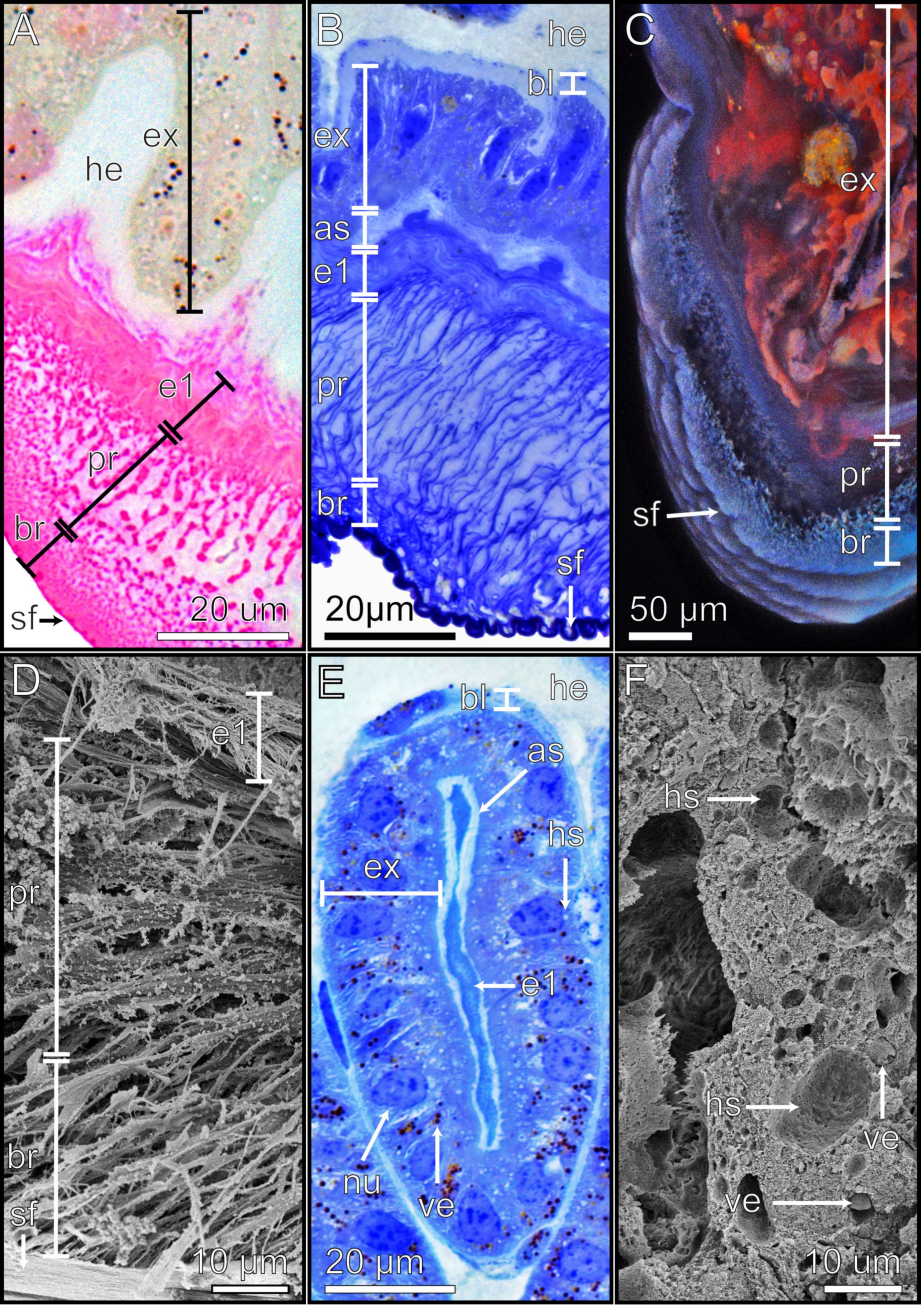
Arolium material structure visualised using different techniques. Detailed images of the adhesive pad of the arolium. The different methods highlight the morphological and structural characteristics of the respective layers and structures. (A) Light microscopy image of the cross section stained with the Cason triple stain. (B) Light microscopy image of the longitudinal section stained with toluidine blue. (C) CLSM image of the cross section. (D) SEM image of the cross section. (E) Light microscopy of the longitudinal section of the exocrine cells stained with toluidine blue. (F) SEM image of the cross section of the exocrine cells. The ventral side of the arolium is oriented towards the bottom of the pictures. As = adhesive secretion reservoir; bl = basal layer; br = branching rod layer; e1 = endocuticle layer 1; ex = exocrine cells; he = hemolymph; hs = hollow spaces; nu = nucleus; pr = primary rod layer; sf = superficial layer; ve = vesicles.

The internally located ≈10 µm wide endocuticle layer 1 (e1) is characterized by its loose, parallel arrangement of sheets, which are discernible through their red staining with Cason (Figure 3A) and blue staining with toluidine blue (Figure 3B). This parallel arrangement is also evident in SEM (Figure 3D) and in longitudinal microtome sections in the light microscope (Figure 3B). In CLSM, the endocuticle layer 1 exhibits a relatively low degree of sclerotization (Figure 3C).

On top of the endocuticle layer 1 there is a ≈30 µm thick primary rod layer (pr) consisting of wide rods extending towards the surface of the arolium and branching into finer rods forming another ≈10 µm thick branching rod layer (br) (Figure 3). The primary rod layer and the branching rod layer are notably stained red by Cason stain (Figure 3A) and blue by toluidine blue stain (Figure 3B), confirming their cuticular origin. The CLSM images further revealed that both layers emit a blue signal, indicative of the presence of resin (rubber-like protein) with relatively soft properties (Figure 3C). The morphological details of these layers are also apparent in longitudinal microtome sections (Figure 3B) and SEM sections (Figure 3D). The primary rod layer is comprised of relatively thick cuticle fibres that branch into finer ones within the branching rod layer, terminating in the superficial layer (sf) (Figure 3D).

The superficial layer is the outermost layer in the arolium and is in direct contact with the environment. When examined with a light microscope, this layer appeared remarkably smooth. Notably, Cason staining resulted in a deep red hue, while toluidine blue staining resulted in a dark blue coloration (Figure 3A, Figure 3B), indicating that the superficial layer consists of a more densely packed cuticle if compared to the rods of the primary rod layer and branching rod layer. Additionally, the cuticle of the superficial layer displays a low degree of sclerotization as indicated by CLSM results (Figure 3C).

### Arolium exocrine cells

The exocrine cells (ex) of the epidermal cell layer are separated from the hemolymph reservoir (he) by a basal layer (bl) which is stained light blue by toluidine blue. (Figure 3B, Figure 3E). The identification of exocrine cell bodies is facilitated by their blue coloration when stained with toluidine blue (Figure 3B, Figure 3E), alongside the presence of a thick basal lamina and numerous discernible cellular structures. When observed in CLSM, the exocrine cells exhibit a red autofluorescence signal (Figure 3C). Notably, the exocrine cells possess large nuclei (nu) with multiple nucleoli, which are prominently stained in shades of blue by toluidine blue (Figure 3B, Figure 3E). Light microscopy revealed the presence of numerous vesicles (ve), which can be distinguished as either black when stained with toluidine blue and Cason or show an orange colour without staining (Figure 3A,B,E). When observed using SEM, these vesicles appear smooth and appear to be detached from the surrounding cellular structures (Figure 3F). Furthermore, round and unstained areas were observed (Figure 3A,B,E). When examined in SEM, these structures appear as hollow, empty spaces (Figure 3F). These structures are named hollow spaces (hs). Based on all these characteristics, the exocrine cells of the epidermal cell layer are likely classified as exocrine cells type I [62].

The basal and apical sides of the exocrine cells exhibit surface expansions towards the basal layer (basal) and the adhesive secretion reservoir (as) (apical) (Figure 3B). The adhesive secretion reservoir is stained light blue with toluidine blue and is situated between the exocrine cells and the epicuticle layer 1 (Figure 2A; Figure 3B).

### Tarsomere structure

Only tarsomeres that possess an attachment pad (euplantulae) were examined and are described below. These tarsomeres measure ≈330 µm in length and ≈210 µm in width (depending on the tarsomere).

**Figure 4:**
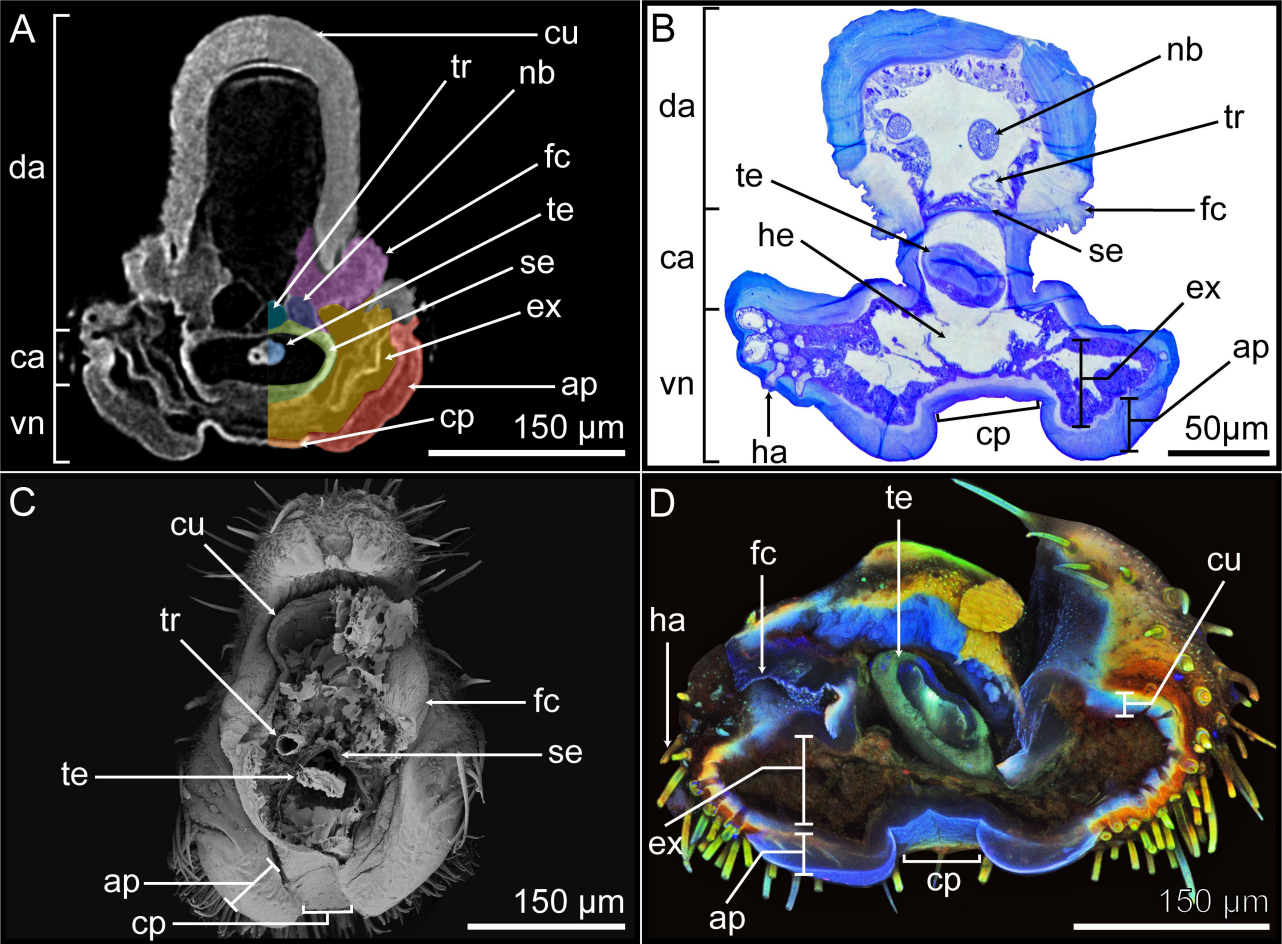
Morphology of the tarsomere. The internal ultrastructure of the tarsomere was visualized using four different methods, which show the different layers and highlight their morphological and structural characteristics. The following methods were used: (A) µCT image of the cross section. (B) Light microscopy cross section stained with toluidine blue. (C) SEM overview of the entire tarsomere. (D) CLSM cross section of the tarsomeres. The ventral sides of the euplantulae are oriented towards the bottom of the images. The examined sections originate from individual tarsomeres along the tarsus, whereby the length and width proportions can differ. ap = attachment pad; ca = central area; cp = connective pad; cu = sclerotized cuticle; da = dorsal area; ex = exocrine cells; fc = flexible cuticle; ha = hair/seta; he = hemolymph; nb = nerve bundle; se = septum; te = tendon; tr = trachea; vn = ventral area.

The septa (se) separate the interior of the tarsomere into four sections. Two thin septa laterally segregate it into two areas on the ventral side (vn), while a comparably thicker septa separates the tarsomere into central and dorsal areas. The central area (ca) accommodates the tendon (te), and the dorsal area (da) the tracheal structures (tr) and nerve bundles (nb). Notably, each of these areas possesses an individual hemolymph channel for circulatory and possible structural purposes through hydrostatic pressure (Figure 4). The septa are dyed blue by toluidine blue and show a parallel cuticle layering in SEM (Figure 4B, Figure 4C).

The cuticle on the ventral part of the dorsal area shows distinctive morphological and structural characteristics compared to the rest of the cuticle, as it lacks the typical toluidine blue staining and autofluorescence of the sclerotized cuticle. In contrast, the region is stained light blue with toluidine blue and exhibits a low degree of sclerotization in CLSM. Moreover, it presents a unique morphology, appearing fanned out, suggesting a more flexible structure (Figure 4B, Figure 4D). Based on these characteristics, this cuticle region is named flexible cuticle (fc). The µCT imaging of the ventral side of the euplantulae revealed a dense hull (lighter grey) and a more X-ray transparent body (darker grey) (Figure 4A). Toluidine blue staining detected a darker blue stained hull and a lighter blue body (Figure 4B). The SEM images unveiled a rather smooth surface topography (Figure 4C). Furthermore, CLSM detected a weak degree of sclerotization (blue autofluorescence signal) of the whole structure (Figure 4D). All these features indicate that this ventral structure is the euplantula attachment pad (ap) that makes direct contact with the substrate. The attachment pad is ≈60 µm wide and laterally merges with the sclerotized cuticle of the tarsomere. This is recognizable by the different coloration of the lateral exoskeleton which shows the staining by toluidine blue and CLSM autofluorescence wavelength signals typical for the sclerotized cuticle (Figure 4B, Figure 4D). The attachment pads of the tarsomeres internally extend into the corresponding tarsomere.

The structure connecting the two attachment pads shows morphological similarities with the attachment pad. In the µCT, the outer hull of this structure exhibits high radiodensity and the inner body shows lesser density (Figure 4A). Similarly, light microscopy with toluidine blue staining showed the outer hull in dark blue and the inner body in a lighter shade of blue (Figure 4B). The SEM images revealed a smooth surface (Figure 4C), while CLSM analysis demonstrated a low degree of sclerotization, suggesting the presence of soft cuticle (Figure 4D). Due to these morphological similarities and the fact that this structure connects the attachment pads, it is referred to as a connective pad (cp).

On the internal side of both the attachment pad and connective pad*,* an epidermal cell layer is situated. This layer encompasses the entire surface of the ventral interior of the tarsomeres, restricting the hemolymph reservoir inside. The layer is separated from the remaining tarsomere tissue by septa. The epidermal cells appear radiolucent in the µCT cross sections (Figure 4A) and are stained blue with toluidine blue (Figure 4B). Also, they show a weak green autofluorescence signal in CLSM (Figure 4D). These findings indicate that the epidermal cell layer consists of exocrine cells (ex). Furthermore, the lateral sides of the tarsomeres exhibited discernible nerve bundles and hair/seta attachment sites (ha), extending into the epidermal layer (Figure 4B).

### Euplantula ultrastructure

**Figure 5:**
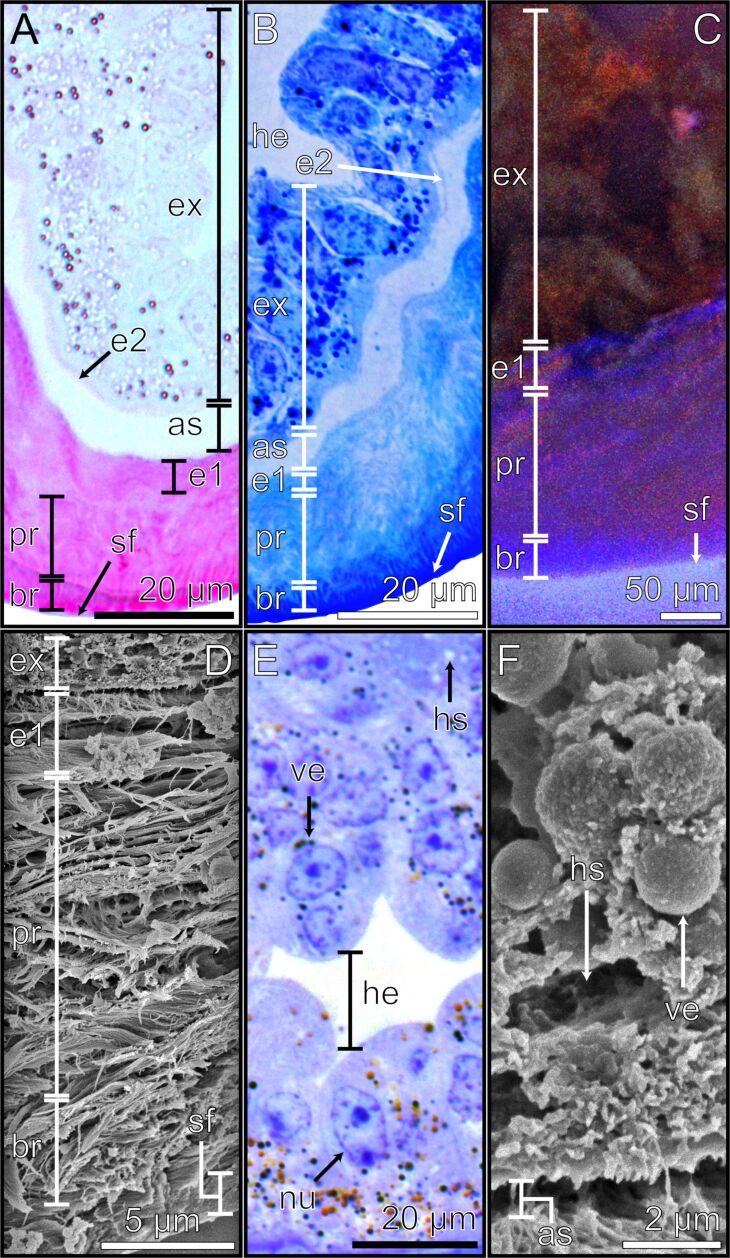
The euplantula sections. Detailed images of the attachment pad of the euplantula. The different methods highlight the morphological and structural characteristics of the respective layers and structures. (A) Cross section stained with Cason’s stain, light microscopy. (B) Longitudinal section stained with toluidine blue, light microscopy. (C) Cross section in CLSM. (D) Cross section in SEM. (E) Longitudinal section of the exocrine cells stained with toluidine blue, light microscopy. (F) Cross section of the exocrine cells in SEM. The ventral side of the euplantulae is oriented towards the bottom of the images. as = adhesive secretion reservoir; br = branching rod layer; e1 = endocuticle layer 1; e2 = endocuticle layer 2; ex = exocrine cells; he = hemolymph; hs = hollow spaces; nu = nucleus; pr = primary rod layer; sf = superficial layer; ve = vesicles.

The inner layer of the attachment pad (ap) is ≈1.5 µm wide, stained light red and blue by Cason and toluidine blue, respectively (Figure 5A, Figure 5B), exhibiting a low degree of sclerotization in CLSM (Figure 5C) and composed of parallel layers of cuticle sheets (Figure 5D). This composition identifies the layer as the endocuticle layer 1 (e1).

From the endocuticle layer 1 emerges a ≈12 µm thick layer of dense wide rods, which subsequently ventrally branches towards the surface into finer, denser rods, and finally terminate into a ≈4 µm thick superficial layer (sf) (Figure 5A,B,D). The layer composed of thick rods is the primary rod layer (pr) and the layer with the finer rods is the branching rod layer (br) (Figure 5D). Cason and toluidine blue staining resulted in a lighter red and blue coloration, respectively, for the cuticle of the primary rod layer compared to that of the branching rod layer, likely reflecting the denser fibrous structure of the latter (Figure 5A, Figure 5B). The CLSM analysis revealed a low degree of sclerotization in both layers, suggesting soft cuticle, with discernible regions of reddish autofluorescence signals, possibly attributed to residual adhesive secretions within the cuticle layers, or to underlying epidermal cells (Figure 5C).

The finer fibers of the branching rod layer ultimately terminate in the superficial layer (Figure 5A,B,D). The thin superficial layer is the outermost layer of the euplantulae, establishing direct contact with the substrate (Figure 5D). Examination in the light microscope and SEM revealed a smooth surface of the pad (e.g., Figure 4C). Staining with Cason and toluidine blue resulted in a dark red or dark blue hue, respectively, indicative of a tightly packed cuticle (Figure 5A, Figure 5B). Additionally, CLSM unveiled a low degree of sclerotization in the superficial layer (Figure 5C).

### Euplantulae exocrine cells

The hemolymph reservoir (he) is ventrally surrounded by a layer of epidermal cells. The basal region of this layer establishes direct contact with the hemolymph with evaginations increasing the contact surface area (Figure 5B). When stained with toluidine blue or Cason, the epidermal cell layer displays deep blue and light red colorations, respectively (Figure 5A,B,E). In CLSM, the layer exhibited a strong green signal with weak red signal portions (Figure 5C). The cells within the epidermal layer house a prominent nucleus with multiple nucleoli, stained in a deeper blue and red by the two staining methods, respectively (Figure 5A,B,E). Due to these characteristics, the cells within the epidermal layer are identified as exocrine cells (ex). Additionally, light microscopy images revealed vesicles (ve) inside the cells. These either exhibited substantial staining intensity due to the applied staining methods or displayed an orange coloration without staining (Figure 5A,B,E). Upon examination through SEM, they appeared spherical and presented either a smooth or slightly rough surface (Figure 5F). Within the exocrine cell layer, unstained larger hollow spaces (hs) were observed (Figure 5A,B,E). Examination via SEM revealed these hollow spaces to appear within the exocrine cell layer, after chemical fixation and critical point drying (Figure 5F). These morphological characteristics identify these cells within the euplantulae as exocrine cells type I [62]. The exocrine cells are enveloped and separated on the ventral side by a thin cuticle layer. This layer is notably more intensely stained in blue (toluidine blue) and red (Cason) compared to the exocrine cells, and is identified as the endocuticle layer 2 (Figure 5A, Figure 5B). The endocuticle layer 2 ventrally borders a ≈2 µm wide layer which runs along the entire length of the attachment pad and the connective pad, laterally terminating into the sclerotized cuticle of the tarsomeres. This layer is very lightly stained by toluidine blue and Cason (Figure 5A, Figure 5B) and named adhesive secretion reservoir (as).

### Connective pad

**Figure 6:**
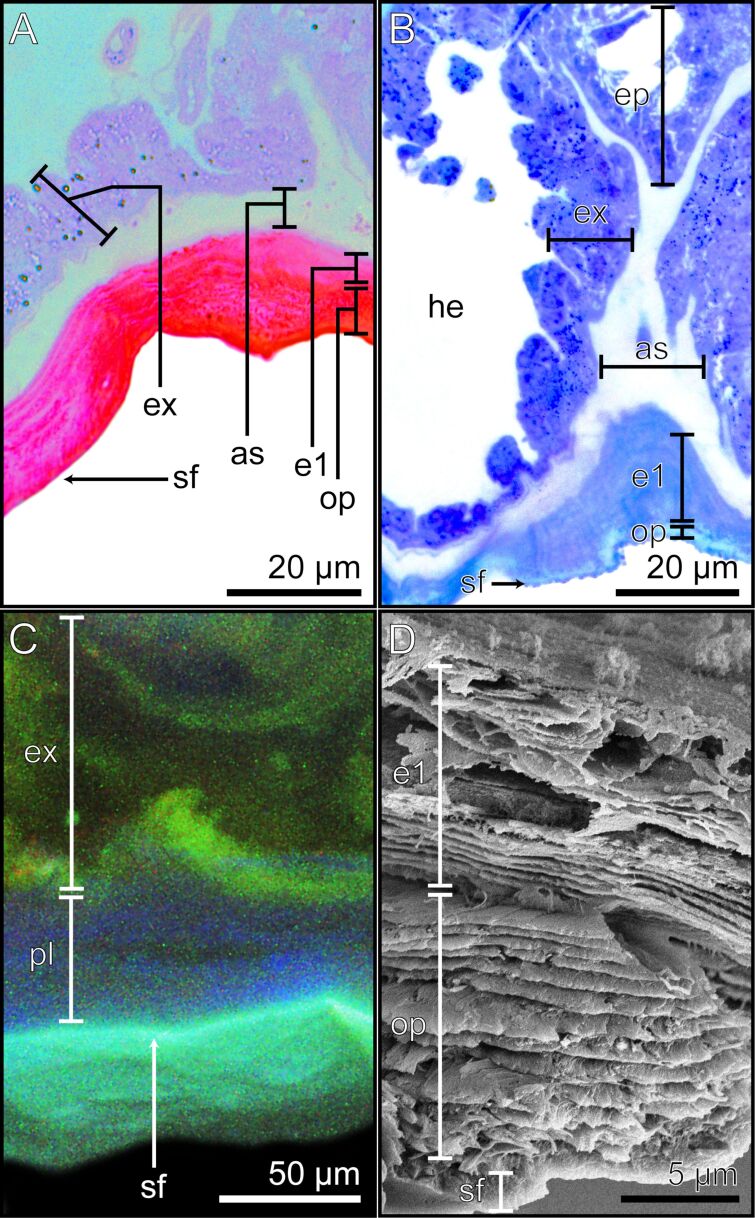
The connective pad between neighbouring euplantulae. Detailed images of the connective pad. The different methods highlight the morphological and structural characteristics of the respective layers and structures. (A) Cross section of the connective pad stained with Cason’s stain, light microscopy. (B) Longitudinal section of the connective pad was stained with toluidine blue, light microscopy. (C) Cross section in the CLSM. (D) Cross section in the SEM. The ventral sides of the connective pads are oriented towards the bottom of the images. as = adhesive secretion reservoir; e1 = endocuticle layer 1; ex = exocrine cells; op = outer parallel layer; pl = parallel layer; sf = superficial layer.

The connective pad medially connects the two euplantulae (Figure 4A, Figure 4B; Figure 6A, Figure 6B). The ultrastructure of the connective pad comprises two layers of parallel cuticle sheets with a ventral terminating superficial layer (sf). The adhesive secretion reservoir and exocrine cells of the euplantulae internally extend and connect the tissues of the two euplantulae (Figure 5). The two parallel cuticular layers are distinguishable in terms of coloration through Cason and toluidine blue staining. The layer situated dorsally adjacent to the adhesive secretion reservoir, exhibited a light red hue stained with Cason and a light blue hue with toluidine blue, identifying it as the endocuticle layer 1 (e1). The outer layer presented a more intense coloration identifying it as the outer parallel layer (op) (Figure 6A, Figure 6B). The CLSM analysis indicates a blue indistinguishable autofluorescence signal in both layers, indicating their low degree of sclerotization (Figure 6C). The SEM images revealed structural similarities between the two layers, with the outer parallel layer displaying a slightly denser layering (Figure 6D). The morphology of the superficial layer in the connective pad corresponds to the characteristics of the superficial layer in the attachment pads, exhibiting a more intense staining with Cason and toluidine blue (Figure 6A, Figure 6B), a low degree of sclerotization (Figure 6C), and a dense cuticle organization, evident via SEM, than that of the outer parallel layer and endocuticle layer 1 (Figure 6D). Both the exocrine cells and the adhesive secretion reservoir of the connective pad exhibit the same morphological characteristics as those of the attachment pads (Figure 5; Figure 6).

### Additional morphological observations

**Figure 7:**
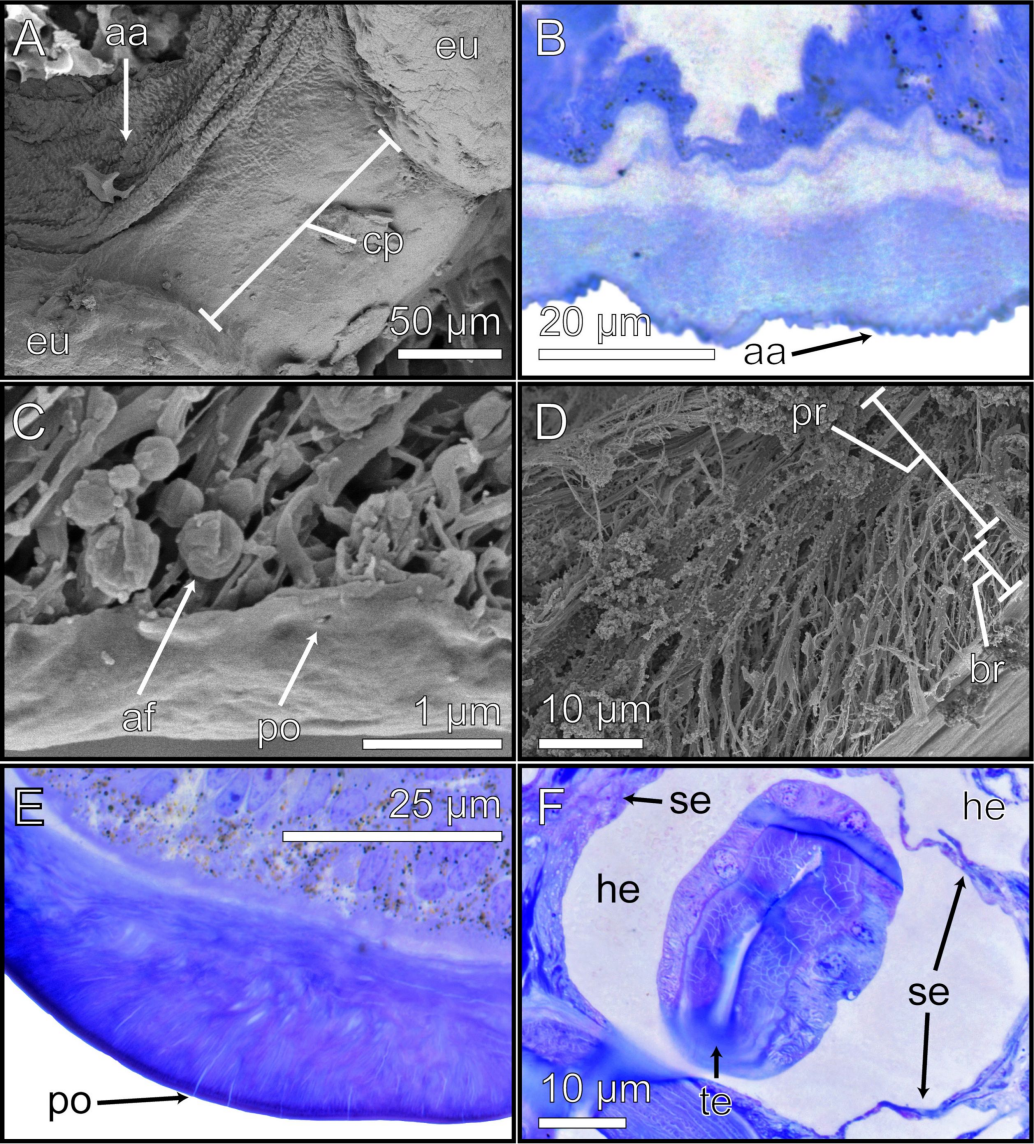
Detailed images of additional morphological observations. The different methods highlight the morphological and structural characteristics of the respective layers and structures. (A) The top view on the euplantulae of one tarsomere shows the connective pad, SEM. (B) Longitudinal section of the connective pad was stained with toluidine blue, light microscopy. (C, D) Cross sections of the arolium, SEM. (C) Superficial layer, SEM. (D) Primary rod layer and branching rod layer, SEM. (E) Longitudinal section of the euplantula stained with toluidine blue, light microscopy. (F) Cross section of the euplantula stained with toluidine blue, light microscopy. aa = anti-adhesive structures; af = adhesive fluid; br = branching rod layer; cp = connective pad; he = hemolymph; po = pore opening; pr = primary rod layer; se = septum; te = tendon.

The superficial layer of the connective pad bears distinctive spherical shapes, which are situated on the dorsal ridges of the connective pad in proximity to the central region of the tarsomere (Figure 7A, Figure 7B). These putatively anti-adhesive structures (aa) were also discovered on the dorsal edge of the arolium (Figure 3B). The SEM and light microscopy (toluidine blue staining) images revealed pore openings (po) in the superficial layer of the euplantulae (Figure 7C, Figure 7E). In addition, small spherical bodies were observed throughout the primary rod layer and branching rod layer, as well as directly beneath the superficial layer of the euplantulae and were identified as adhesive fluid residues (as) (Figure 7C, Figure 7D).

## Discussion

### Similarities between the two attachment pad types

The anatomy and material composition of the two tarsal attachment organs, euplantulae and arolium, were compared using different imaging techniques. The study revealed some similarities between them, corresponding to their roles in the attachment process [1]. In the interior of both organs, there is a hemolymph reservoir serving dual purposes as a hydrostatic support system and a supply of nutrients to the cells [63]. Following the hemolymph reservoir, exocrine cells are present in the epidermal layer of both organs. As transformed epidermal cells, the exocrine cells are responsible for the secretion of all cuticular layers apical to them, as well as the production of the adhesive secretion. These layers encompass the endocuticle layers 1 and 2, the adhesive secretion reservoir, the primary and branching rod layers, as well as the superficial layer [41,64]. These exocrine cells exhibit surface extensions into the hemolymph and adhesive secretion reservoir optimizing the substance absorption and discharge [31,65–67]. Adjacent to the exocrine cells is the adhesive secretion reservoir serving for the accumulation of the produced adhesive secretion. Both pad types share a similar organisation of the procuticle. The endocuticle layer 2 has a parallel cuticle layering, the primary rod layer is composed of wide cuticle rods ventrally branching into finer rods within the branching rod layer, terminating in the superficial layer (Figure 3; Figure 5).

Previous investigations of the smooth attachment pads (arolium and euplantulae) of *Gromphadorhina portentosa* (Schaum, 1853) by Schmitt and Betz [45] revealed a similar layering of both attachment pads. Similar structures of the procuticle, especially the primary rod-*,* branching rod-, and superficial layer were also reported by Gorb et al. [20], Gorb and Scherge [21], and Goodwyn et al. [44] in the smooth euplantulae of *Tettigonia viridissima* (L., 1758) and *Locusta migratoria* (L., 1758). Differences in the layering and the details of microstructure likely evolved due to variations in their ecological lifestyle.

Several insects possess hairy attachment organs, which morphologically differ from the smooth ones examined herein. The differences between them manifest primarily in the morphology of the procuticle region. Hairy attachment organs are characterized by cuticle outgrowths (e.g., setae or acanthae [5,68–71]), whereas smooth attachment organs consist of hierarchically split cuticle rods terminating in the superficial layer creating a rather smooth surface [20,70–71]. Both types of attachment organs utilise their distinct morphologies to efficiently replicate the substrate profile to a similar extent, thereby amplifying the actual contact area and, consequently, enhancing attachment [1–2,72–73].

### Differences between smooth and hairy attachment pads

The primary difference between hairy and smooth attachment organs manifests in the cuticular morphology. Hairy attachment organs consist of cuticle outgrows (e.g., setae or acanthae [5,68–70]), the cuticle of smooth attachment organs consists of filaments that hierarchically split terminating in the superficial layer, creating a rather smooth surface at the level of light microscopy [20,70–71].

Both types of attachment pads efficiently replicate the surface profile of the substrate owing to their distinct structures, thereby augmenting the actual contact area and, consequently, enhancing attachment. Smooth attachment pads accomplish this through hierarchical organization and the viscoelastic properties of the cuticle [1–2,72–73].

### Differences between the two attachment pad types

Despite the similar overall morphology, the two attachment organs show some distinct structural differences, which can be attributed to different functions that both types fulfil. Previous research on the attachment pads of the phasmid *Carausius morosus* (Brunner von Wattenwyl, 1907) and the cockroach *Nauphoeta cinerea* (Olivier, 1789) proposed that the arolium primarily serves to generate adhesion, while the euplantulae predominantly function for the generation of friction, characterizing the arolium as an adhesive pad and the euplantulae as friction pads [52–54]. Adaptation to the specific requirements is realized in euplantulae and arolia by the different morphological organizations.

### Primary rod layer and branching rod layer

In the arolium, the fibres in the primary rod layer and branching rod layer are notably thicker and more widely spaced compared to those in the euplantulae (Figure 3 (arolium); Figure 5 (euplantulae)). In general, the hierarchical organization of the fibres enables local deformation to adjust to the surface profile of the substrate (e.g., [20,45,70]). This results in anisotropic material properties (i.e., the pads are soft during compression); however, those withstand high tensile stress [74–75]. The more spaced fibres of the arolium consequently would bend more efficiently under pressure and easily adapt to surface irregularities increasing adhesion [44]. The euplantulae feature relatively thinner and with more densely distributed fibres enhances protection against environmental conditions such as wear [76] and evaporation [44]. This enhanced resilience comes at the expense of reduced adaptability to surface irregularities. As a frictional pad, the euplantula requires increased wear resistance, prioritizing it over optimal conformability to surfaces to withstand applied shear forces without undergoing degradation.

Similar morphological features have been previously described by Clemente and Federle [54] for the arolium and euplantulae of the cockroach *N. cinera*, by Bennemann et al. [71] for the arolium of the stick insect *C. morosus*, and by Schmitt and Betz [45] for the arolium and euplantulae of the cockroach *G. portentosa*.

The hollow spaces between fibres within the primary rod layer and the branching rod layer can also be important for adjusting the material properties of the attachment pads. Adhesive secretion kept within the spaces could impact the viscoelasticity of the pad, as well as its shape due to the internal pad pressure caused by the fluid. Spherical structures between the fibres, identified via SEM, could be indications for liquid residues (Figure 7C) (similar residues have been also identified by Gorb et al. in the euplantulae of *T. viridissima* [20]). In addition, the red CLSM autofluorescence signal within the euplantulae might have been caused by the adhesive fluid or by the exocrine cells (Figure 5C), assuming it contains organic molecules with a conjugated system of electrons caused by C=C double bonds [39–40]. The adhesive secretion within the primary rod layer and branching rod layer could work as a soft backing enhancing the conformability to the substrate and friction generation in contact with rough substrates [77].

### Endocuticle layer 1

Another morphological difference between the arolium and euplantulae is observed in the endocuticle layer 1. In the arolium, the endocuticle layer 1 is thicker (arolium: ≈10 µm; euplantula: ≈5 µm) (Figure 3; Figure 5D) and more intensely stained with toluidine blue and Cason compared to that of the endocuticle layer 1 of the euplantulae (Figure 3; Figure 5). This difference potentially arises from the larger volume of the arolium, necessitating a stronger endocuticle layer 1 as a support for the primary and branching rod layers. Additionally, the parallel layer structure of the endocuticle layer 1 could give additional resistance against shear forces [78].

### Exocrine cells

The exocrine cells of both attachment pads show multiple morphological similarities. Both exocrine cells display comparable staining patterns with toluidine blue and Cason. They possess a sizable nucleus containing numerous nucleoli, a substantial abundance of vesicles and hollow spaces, the absence of a discernible structural mechanism for product release (e.g., a duct), and the presence of a dedicated storage area for their respective products (e.g., the adhesive secretion reservoir).

Collectively, these distinctive features categorize the exocrine cells as exocrine cells type I [62]. Despite their morphological similarities, there are a few differences between the exocrine cells of the arolium and the euplantulae. The initial distinction is the presence of a wide basal layer in the arolium situated between the exocrine cells and the hemolymph (Figure 3; Figure 5). Although the euplantulae likely possess a very thin basal layer, similar to that found in *G. portentosa* [45], confirmation requires TEM analysis. The presence of the wide basal layer potentially augments the mechanical stability of the exocrine cells and ultimately of the arolium [79].

Another difference lies in the autofluorescence of both exocrine layers in CLSM. The exocrine cells of the arolium exhibit a stronger red autofluorescence signal, while those of the euplantulae display green autofluorescence (Figure 3 (arolium); Figure 5 (euplantulae)). Both attachment pads were separately scanned but under the same conditions and settings. Therefore, the difference in the autofluorescence signal could be the result of the two scans (i.e., surrounding material influencing the projected intensity) or be an indication of a difference in composition between the two cell aggregations.

Morphological investigations of the adhesive fluid of *M. extradentata* using cryo-SEM revealed different structures that the fluid can adopt, as well as slight differences between those of arolium and euplantulae [38]. It was postulated that these structures arise due to different mixing ratios of the fluid, and that the fluid can therefore fulfil different functions.

Our results remain ambiguous. The morphological similarities between the exocrine cells of both types of pads suggest that both produce the same adhesive fluid, which is potentially differentiated by various mixing ratios or production rates. It is also possible that the difference in the autofluorescence indicates that the arolium and euplantulae produce different substances.

Schmitt and Betz [45] discovered no major morphological differences between the exocrine cells of the arolium and euplantulae of *G. portentosa* as well. This could be an indication that the adhesive fluid and its production may be similar between the two species.

Furthermore, the exocrine cell layer of the arolium is more strongly folded in comparison to that of the euplantulae (Figure 2 (arolium); Figure 4 (euplantulae)). The enlarged surface could offer more exocrine cell area increasing the discharge area of the secretion, as well as allowing the pad to deform more easily, making it more resistant to mechanical stresses.

### Endocuticle layer 2

The endocuticle layer 2 is strongly pronounced around the exocrine cells of the euplantulae, as evidenced by the darker staining with toluidine blue and Cason (Figure 5A, Figure 5B). In the arolium, however, the endocuticle layer 2 is not recognizable.

The wider endocuticle layer 2 in the euplantulae could be a structural feature that increases the resistance to shear forces as well as the stability of the attachment organ. A layer with similar properties, the inner cuticular band, has been previously observed in the arolium and euplantulae of *G. portentosa* by Schmitt and Betz [45].

### Adhesive secretion reservoir

The endocuticle layer 1 and endocuticle layer 2 are separated by a confined space measuring ≈10 µm in width, the adhesive secretion reservoir, which is slightly stained with toluidine blue and Cason in both the arolium and euplantulae (Figure 3; Figure 5). Based on the light staining with toluidine blue and Cason, the adhesive secretion reservoir probably consists of very loosely packed cuticle fibres which allow the adhesive secretion to be stored. Due to the potentially loose structure of the adhesive secretion reservoir, it is susceptible to rupture, whereby the actual size of the reservoir is difficult to determine. In addition to serving as a repository for the secretion, this reservoir could play an additional role in providing a pliant support structure when filled with adhesive secretion, thereby contributing to the stabilization of the respective attachment pad [77]. A morphologically similar adhesive secretion reservoir layer was also observed in the arolium and euplantulae of *G. portentosa*, as well as in the arolium of *T. viridissima* [45,80].

### Internal subdivision of the euplantulae

The division of the euplantulae into four areas (Figure 4; Figure 7F) results in four independent volumes filled with hemolymph capable of generating internal hydraulic pressure. This pressure could potentially influence the shape of the euplantulae and therefore control the attachment process. Similar principles were discovered in the toe pads of tree frogs where the blood pressure maintains its shape [81], and in the arolia of ants where hemolymph pressure inflates them [82]. Dening et al. [83] showed in an artificial system that internal air-filled bladders can control attachment strength.

### Anti-adhesive structures

The superficial layer of the connective pad is patterned in a hemispherical shape at the predominantly peripheral position towards the centre. This position suggests that such structures act as an adhesion- and friction-reducing system (anti-adhesive structures, Figure 7B) [86]. The hemispherical pattern reduces the contact area between the cuticle and the substrate, thus decreasing contact forces. Similar surface structures were observed in the wax coverage of plants where they decreased the attachment performance of insects [84]. A reduction of the contact area and the resulting reduced adhesion was shown by Wu et al. [85] for artificial structures. Reducing attachment could be helpful in the areas where such structures are found as they prevent the adhesion of folds in membranous cuticles in the regions of the connective pad. They might also reduce the risk of trapping contaminants in the inter-tarsomeric membranous region. The removal of particulate contaminants is very important as they are known to cause abrasive wear in the open insect joints [86]. Anti-adhesive surface structures in the periphery of the active working areas of the attachment pads could establish zones facilitating detachment. Such detachment movements are described for flies with hairy attachment pads [87], but would function similarly in smooth ones.

### Connective pad

Our investigations have revealed a continuity of several underlying layers from beneath the euplantulae extending through the connective pad region. These layers encompass the hemolymph, exocrine cells, endocuticle layer 2, adhesive secretion reservoir, endocuticle layer 1, and superficial layer (Figure 6). Notably, the connective pad lacks both the primary rod layer and branching rod layer but exhibits an additional stratum of outer parallel cuticle. In CLSM, the exocrine cells exhibit green autofluorescence, whereas the procuticle emits blue autofluorescence, which is consistent with observations in the euplantulae. Footprints of the connective pad revealed residues of the secretory fluid (pers. obs.). Given the identification as a soft cuticle, the structural attributes of the procuticle, and the presence of adhesive secretion, it is possible that the connective pad could participate in the attachment process.

Similar connective pad structures are present in insect species that also possess split euplantulae [88]. However, many euplantulae-bearing insect species do not have split euplantulae and therefore do not possess a connecting tissue [5,45,89–91], including several phasmid species [88,92].

### Transportation pathway of the pad secretion

**Figure 8:**
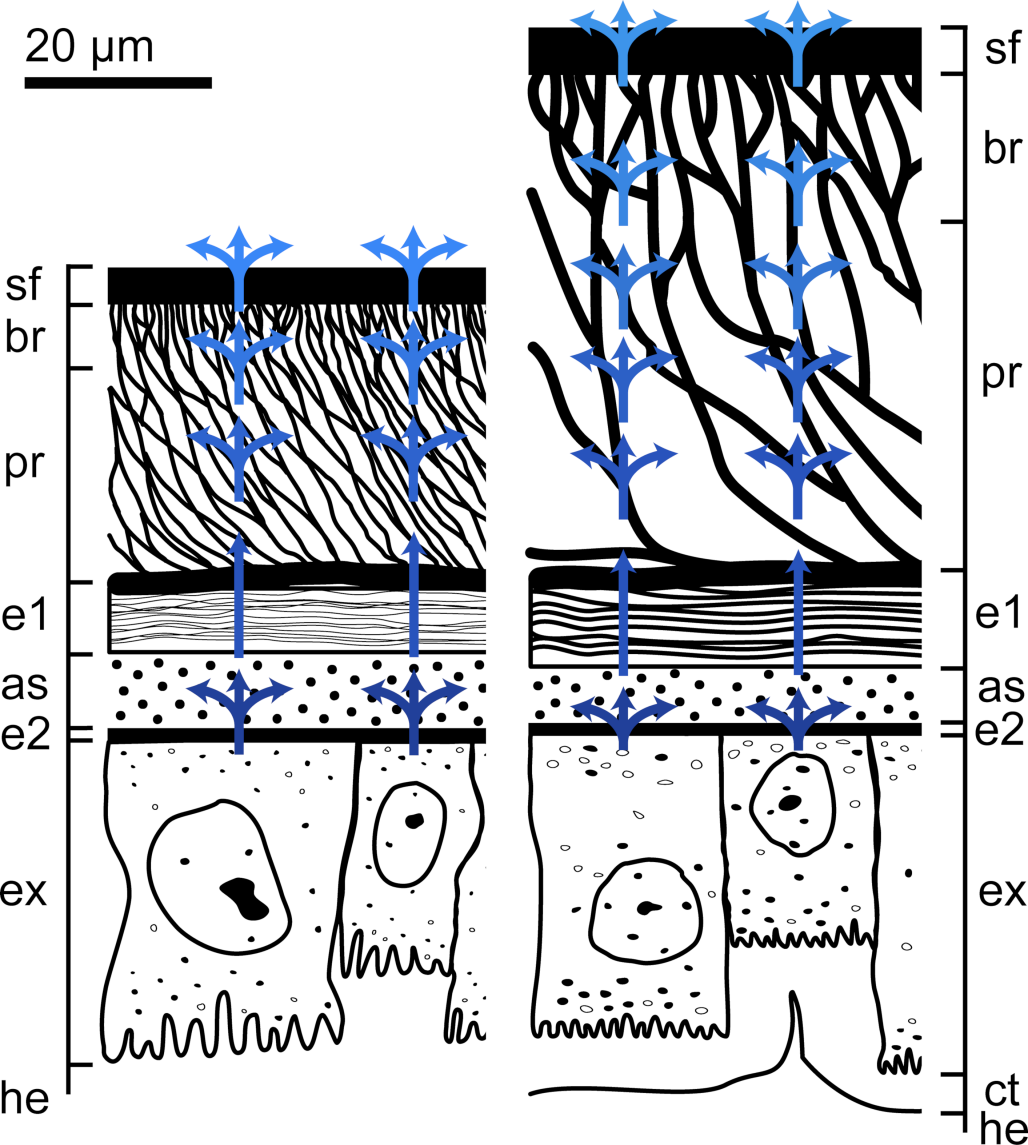
Scheme of the arolium (left) and euplantula (right) of *M. extradentata*. Schematic representation of the transportation of the adhesive secretion from its point of origin towards the substrate. A description of the production and transportation pathway of the adhesive secretion is provided in the text. as = adhesive secretion reservoir; bl = basal layer; br = branching rod layer; e1 = endocuticle layer 1; e2 = endocuticle layer 2; ex = exocrine cells; he = hemolymph; pr = primary rod layer; sf = superficial layer. Blue arrows indicate the pathway of the adhesive secretion in the respective pad type. Brightening of the arrows indicates a reduction in the amount of adhesive fluid.

The schematic representation delineates the potential site of adhesive secretion production and its transportation from the hemolymph reservoir to the surface of the euplantula and arolium (Figure 8). The exocrine cells, situated in the epidermal tissue, obtain the educts for the adhesive secretion from the hemolymph. Both attachment pads exhibit exocrine cells with surface expansions into the hemolymph increasing the area for reactant uptake.

The adhesive fluid is secreted through pores in the endocuticle layer 2 [45] and accumulates in the adhesive secretion reservoir (indicated by the split arrow). Subsequently, the secretion traverses the endocuticle layer 1 via pores [45] and enters the primary rod layer. Within the primary rod layer and branching rod layer layers, the secretion fills the cavities between the rods (indicated by the split arrows), extending throughout the layers up to the superficial layer. The transportation of the adhesive secretion to the surface is facilitated through pores in the superficial layer [64] (Figure 7C,E).

The cuticle layering and morphology of the arolium and euplantulae facilitate the absorption, storage, and distribution of the produced adhesive secretion within the attachment pads, enabling its transport to the surface. As mentioned above, the presence of the fluid secretion in these layers modulates the stability of the corresponding layers, potentially serving as a soft backing enhancing attachment on the substrate by maximizing the contact area [77].

Dirks and Federle [15] observed that the adhesive secretion volume in the phasmid *C. morosus* was completely depleted after approximately 7–10 consecutive press-downs (steps), with a subsequent restoration to its original volume taking approximately 15 min, indicative of a steady-state supply. The existence of multiple reservoirs (the adhesive secretion reservoir as well as the hollow spaces in both the primary rod layer and branching rod layer) suggests a continuous supply of adhesive secretion toward the surface, minimizing the likelihood of complete depletion of the attachment pad. Additionally, the denser cuticle rod structure of the branching rod layer may potentially restrict the flow of adhesive secretion, thereby reducing the risk of excessive fluid production.

Schmitt and Betz [45] also postulated a comparable transport pathway for adhesive secretions in the smooth attachment pads of *G. portentosa*. There, the adhesive secretion produced by exocrine cells type I is transported through a two-layered inner cuticle band via pores (comparable to the endocuticle layer 2) and accumulates in the secretion reservoir. It then passes through a layered cuticle via pores (comparable to the endocuticle layer 1) into a sponge-like cuticle where it fills the hollow cavities (comparable to the primary rod layer and branching rod layer). The final route to the surface is via pores in the ventral cuticle band and the epicuticle (comparable to the superficial layer in this study).

## Conclusion

The examination of the ultrastructure and material composition of the tarsal attachment apparatus of the stick insect *Medauroidea extradentata* yielded insights into the detailed structure of the two attachment pad types (arolium and euplantulae). Our findings revealed differences in the structure and material composition between them, indicative of their different roles during attachment. We proposed a potential pathway for the adhesive secretion from the exocrine cells to the surface and provided evidence suggesting the involvement of exocrine cells type I, which exhibit some variability between the arolium and euplantulae. For a more comprehensive understanding of the functional principles of both pad types, a detailed examination of their ultrastructure and testing of their material properties is required. Transmission electron microscopy and atomic force microscopy are ideal approaches for this purpose.

## Data Availability

All data that supports the findings of this study is available in the published article.
